# Effects of shaving on reproductive performance and protein metabolism in bucks under heat stress conditions

**DOI:** 10.3389/fvets.2026.1784286

**Published:** 2026-07-27

**Authors:** Xiangqian Meng, Zhiqiang Guo, Rui Yang, Yongjun Ren, Jie Zheng, Jincheng Zhong

**Affiliations:** 1 College of Animal Science and Veterinary Medicine, Southwest Minzu University, Chengdu, China; 2 Sichuan Animal Science Academy, Chengdu, China; 3 Animal Breeding and Genetics Key Laboratory of Sichuan Province, Chengdu, China

**Keywords:** heat stress, metabolomics, rabbit, reproductive performance, shaving

## Abstract

**Introduction:**

Heat stress (HS) adversely affects reproductive performance and protein metabolism in rabbits, posing significant challenges to livestock production in high-temperature environments. This study evaluated the effectiveness of summer shaving as a strategy to mitigate HS-induced reproductive impairment.

**Methods:**

Thirty male and 240 female rabbits were assigned to shaved and control groups and exposed to HS conditions (temperature-humidity index, THI ≥ 27.8). Reproductive performance parameters were assessed in males and females. In addition, seminal plasma metabolomics was conducted to investigate metabolic alterations associated with shaving under HS.

**Results:**

Shaving significantly improved semen volume, sperm density, and motility in males and accelerated post-HS recovery. In females, shaving increased litter size, number of live births, and neonatal litter weight under HS, with sustained benefits during the recovery period. Metabolomic analysis revealed distinct metabolic profiles, with upregulation of tryptophan metabolism under normal conditions and tyrosine metabolism under HS, alongside consistent downregulation of oleamide, nucleotide metabolism, and phenylalanine metabolism.

**Discussion:**

These findings suggest that shaving alleviates HS-induced reproductive dysfunction by modulating metabolic pathways, reducing oxidative stress, and promoting resource allocation toward reproduction. As a practical and cost-effective intervention, shaving offers valuable potential for improving rabbit reproductive performance in hot climates.

## 1 Introduction

With the continuous acceleration of global warming and intensified farming environments, heat stress (HS) has gradually become a globally recognized issue in modern livestock and poultry production. HS refers to the collective non-specific responses elicited in animals when exposed to excessively high temperatures beyond their thermoregulatory capacity (1). Heat stress alters various physiological and biochemical responses in the body, leading to physiological dysfunction, reduced production performance, heatstroke, and even death in livestock and poultry (2, 3), resulting in significant economic losses to the livestock industry.

Rabbit farming is an important component of global livestock production. As homeothermic animals, rabbits maintain a normal body temperature of 38.5–39.9 °C, while the upper limit of their thermoneutral zone in all studies is below 30 °C. The optimal growth temperature for meat rabbits ranges from 15 to 25 °C. Due to their dense fur, underdeveloped sweat glands, and primary reliance on respiratory evaporation for heat dissipation, rabbits are particularly sensitive to high-temperature environments (4, 5). When ambient temperatures exceed 30 °C, rabbits are prone to heat stress, and when temperatures surpass 35 °C, they are unable to regulate body temperature, leading to heat exhaustion (6). In terms of reproductive performance, heat stress impairs spermatogenesis in male rabbits by inducing excessive production of reactive oxygen species (ROS) within cells, reducing antioxidant capacity, triggering apoptosis and autophagy of germ cells, and causing DNA damage in mature sperm, thereby reducing sperm quality (7–9). Similar detrimental effects of heat stress on sperm quality, including reduced motility and increased oxidative damage, have been recently documented in dogs, where elevated temperatures disrupt oxidative balance and impair semen parameters (10). In female rabbits, heat stress affects oocyte or follicular maturation and embryonic development, with increased ROS levels similarly causing oocyte damage, thereby reducing fertilization capacity (4, 11). Furthermore, heat stress significantly reduces estrogen secretion in female rabbits, leading to irregular estrus and abnormal oocyte morphology, such as cytoplasmic shrinkage and zona pellucida rupture, rendering oocytes unable to be fertilized, resulting in reduced litter size and lower birth weight (6, 9).

To mitigate the adverse effects of heat stress on rabbits, a proposed strategy is shaving of rabbits during the summer, aiming to enhance heat dissipation efficiency by reducing the insulating layer and improving convective heat loss (12–14). Studies have shown that shearing can lower body temperature and alleviate some heat stress symptoms, with sheared individuals exhibiting lower rectal temperatures, reduced mortality rates of offspring, restored feed intake, and improved feed conversion ratios compared to non-sheared individuals (12, 14), thereby enhancing ketone body traits and reproductive performance under heat stress conditions. However, current studies primarily focus on phenotypic validation, lacking analyses integrated with metabolomics, and the mechanisms by which shaving improves reproductive performance require further investigation. Therefore, this study aims to comprehensively evaluate the effects of summer shaving on alleviating heat stress in rabbits, investigate the impact of shaving on reproductive performance and protein metabolism, and explore its potential regulatory mechanisms using metabolomics techniques, providing a scientific basis for optimizing rabbit farming management.

## 2 Materials and methods

### 2.1 Experimental animals and grouping

Adult male Sichuan White rabbits aged 10–13 months with an average body weight of 2.65 ± 0.22 kg, good physical health and normal reproductive function were selected for the study. After two semen collection training sessions, 30 males with semen quality meeting the minimum criteria (sperm motility ≥10%, sperm viability ≥10%, sperm concentration ≥10 × 10^6^/ml, abnormal sperm rate ≤ 10%, and semen volume ≥0.2 ml) were chosen for subsequent experiments. These qualified males were randomly assigned to either a shaved group or a control group, with 15 rabbits per group. Additionally, 240 adult Sichuan White rabbits with similar body weight and reproductive parity (2–3 litters) were selected as experimental animals, with an average body weight of 2.52 ± 0.17 kg. These reproductively active female rabbits were randomly divided into two groups: a shaved group and a control group, with 120 rabbits in each group. In the shaved group, the fur on the back and outer limbs of the experimental rabbits was clipped to a residual length of 10–15 mm. Before the experiment, the rabbit cages, waterers and troughs in the rabbit house were thoroughly cleaned and fumigated. All experimental rabbits were housed individually in semi-open rabbit sheds under identical feeding and environmental conditionswith ad libitum access to feed and water. The designed crude fiber content of the experimental basal diet was 16%. The other nutrient indexes referred to the recommended nutrient levels proposed by Lebas (15) and the Nutrient Requirements of Meat Rabbits (NY/T 4049–2021) (16). The dietary ingredients were selected and formulated according to the characteristics of feed resources in Southwest China. The composition and nutrient levels of the basal diet are shown in Table 1. The experimental diet pellets had a diameter of 3.2 mm and a length of 6–8 mm. The breeding rabbits used in the experiment were obtained from the breeding rabbit farm of the Sichuan Animal Science Academy.

**Table 1:** Composition and nutritional characteristics of the commercial pelleted feed ration provided to experimental rabbits.

Items	Contents
Ingredients	
Corn (%)	14
Soybean meal (%)	16
Sunflower feed meal (%)	6
Wheat bran (%)	16
Alfalfa (%)	30
Grain chaff (%)	5.5
Rice chaff (%)	9
CaHPO_4_ (%)	0.8
CaCO_3_ (%)	1.2
NaCl (%)	0.5
Premix (%)^a^	1
Total (%)	100.00
Nutrient levels^b^	
DE (MJ/kg)	10.80
CP (%)	17.50
EE (%)	3.12
CF (%)	16.11
Lys (%)	0.81
Met + Cys (%)	0.62
Ca (%)	1.12
P (%)	0.58

^a^The premix provides the following per kg the diet: Fe 70 mg, Cu 20 mg, Zn 90 mg, Mn 20 mg, Mg 110 mg, VA 5,500 IU, VD3 1 500 IU, VE 65 mg, choline 1.0 g;

^b^Nutritional level is measured value.

### 2.2 Temperature-humidity index measurement

An Apresys 179-TH thermo-hygrometer was placed at the center of the rabbit shed, 15 cm above the ground, to record temperature and humidity data at 30-min intervals from August 5, 2024, to October 25, 2024. The temperature-humidity index (THI) was calculated using the formula proposed by Alves et al. (17). to assess the heat stress status of the experimental rabbits: THI < 27.8 indicates no heat stress, 27.8 ≤ THI < 28.9 indicates moderate heat stress, 28.9 ≤ THI < 30 indicates severe heat stress, and THI > 30 indicates extreme heat stress. Temperature, humidity, and THI changes in the rabbit house are presented in Table 2.

**Table 2:** Temperature, humidity, and THI changes in the rabbit house.

Time	Temperature (°C)	Humidity (%)	THI	Heat stress condition
August 5–September 23	29.13 ± 0.30	96.54 ± 3.36	28.97 ± 0.22	Severe heat stress
September 24–October 25	27.83 ± 0.40	97.38 ± 2.48	27.72 ± 0.39	No heat stress

### 2.3 Sample collection

During the heat stress period, semen samples were collected using an artificial vagina from male rabbits in the shaved group and the control group separately, with a collection interval of 2–3 days. Semen from both groups was collected between 8:30 and 9:30 a.m. on the same day, involving 15 male rabbits per group each time. A polyvinyl chloride artificial vagina filled with warm water at 50 °C was used for semen collection. Each male rabbit was collected only once. After visual inspection, ejaculates contaminated with urine or containing calcium carbonate precipitates were excluded. Following preliminary screening, the gelatinous substances in the semen were removed, and each sample was equally divided into two aliquots: one aliquot was diluted with semen extender at a 1:1 volume ratio, stored in a 37 °C constant-temperature incubator for no more than 30 min after collection, and used for semen quality assessment; the other aliquot was centrifuged at 1,500 r/min for 15 min. The separated seminal plasma was stored in 2 ml liquid nitrogen cryotubes for subsequent metabolomic analysis.

### 2.4 Semen quality assessment

The sperm density (concentration) and sperm motility were analyzed using a computer-assisted 145 semen analysis (CASA) system (SpermVision, Minitube, German). The time interval from semen collection to vitality assessment should not exceed 1 hour. Semen volume was determined by weighing with an electronic analytical balance, converting 1 g to 1 ml. Total sperm count was calculated as the product of semen volume and sperm density.

### 2.5 Seminal plasma metabolite extraction

Six seminal plasma samples from each of the shaved and control groups, collected during the heat stress period were thawed at 4 °C and vortexed to mix thoroughly. From each sample, a 100 μl aliquot was combined with 300 μl of pre-chilled methanol. The mixture was incubated at −20 °C for 30 min and then centrifuged at 16,000 × g at 4 °C for 20 min. The supernatant was collected for subsequent LC-MS/MS analysis.

### 2.6 Metabolomics data preprocessing

Raw data were processed using MSDIAL software for peak alignment, retention time correction, and peak area quantification. Data analysis was conducted after metabolite structure identification and quality control. To explore potential metabolic pathways, public databases, including HMDB, MassBank, and GNPS, along with an in-house BaiPu metabolite database (BP-DB), were employed.

### 2.7 Determination of reproductive performance in female rabbits

A total of 240 multiparous female rabbits (120 rabbits per group) were used in this study. Fresh semen was collected from male Sichuan White rabbits raised on the farm. After passing semen quality evaluation, the semen was isothermally diluted at 30–35 °C and immediately used for artificial insemination. Each doe was inseminated once with a volume of 0.5 ml, containing at least 2 × 10^7^ viable spermatozoa per dose. Artificial insemination was performed using disposable curved-tip catheters between 8:00 and 9:00 a.m. To induce ovulation, 0.5 ml of luteinizing hormone-releasing hormone (LHRH) was intramuscularly injected into the inner thigh of each doe at the time of insemination. Prior to insemination, the does were subjected to estrous synchronization for 6 days by controlling the photoperiod (16 h of light per day, light intensity 90 lx). The following parameters were recorded for each group: conception rate, litter size, number of live kits per litter, initial litter weight, and litter weight at 21 days.

### 2.8 Statistical analysis of data

The raw data were statistically processed using Excel and the data were analyzed using univariate analysis of variance (T-test) in Graphic Prism 10.1.2 software. First, the raw mass spectrometry files were converted to mzXML format using the MSConvert tool within the Proteowizard software package (v3.0.8789). Peak detection, filtering, and alignment were performed using the R XCMS software package (v3.12.0) to obtain metabolite quantification data. Identify substances using public databases such as HMDB, MassBank, LipidMaps, mzCloud, KEGG, and other spectral databases. The R package Ropls was employed to perform dimensionality reduction analysis on the sample data using principal component analysis (PCA), partial least squares discriminant analysis (PLS-DA), and orthogonal partial least squares discriminant analysis (OPLS-DA). The models were tested for overfitting using the permutation test method. Differentially metabolized compounds were screened using VIP > 1 and P < 0.05.

## 3 Results

### 3.1 Effects of shaving on reproductive performance of male bucks under heat stress

**Figure 1:**
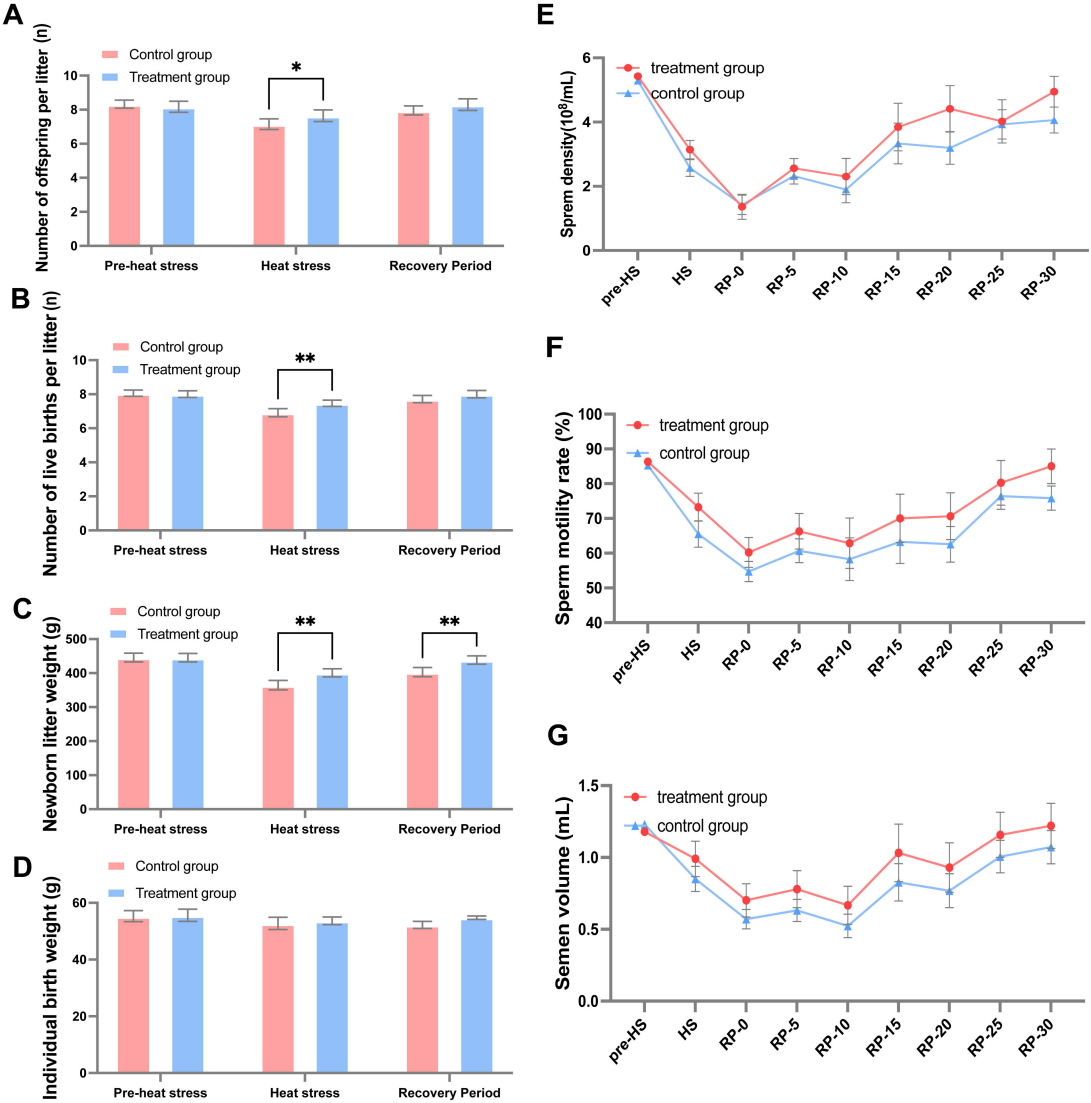
The effects of shaving in female and male rabbits under heat stress conditions. Treatment group: shaved. Control group: unshaved. **(A–D)** illustrate the effects of HS on the following reproductive performance indicators in female rabbits: **(A)** Number of offspring per litter. **(B)** Number of live birth litter. **(C)** Newborn litter weight. **(D)** Individual birth weight. **(E–G)** illustrate the effects of HS on the following reproductive performance indicators in male rabbits: **(E)** Semen volume. **(F)** Sperm density. **(G)** Sperm motility rate. RP-0~RP-30: the corresponding data recorded by the recovered male rabbits every 5 days.

The effects of shaving on the reproductive performance of male rabbits under heat stress were evaluated by comparing the treatment group (shaved) with the control group (unshaved) over a 30-day recovery period following HS. As shown in Figure 1, several key parameters including semen volume, sperm density, and sperm motility rate were determined at designated intervals (RP-0 to RP-30). Initially, under heat stress, the treatment group exhibited significantly higher semen volume, sperm density, and sperm motility compared to the control group (Figures 1E–G). Throughout the recovery period, both groups showed fluctuating improvements in these parameters; however, treatment groups recovered to pre-heat stress levels more rapidly compared to control groups.

### 3.2 Effects of shaving on reproductive performance of female rabbits under heat stress

In pregnant female rabbits, prior to heat stress (pre-HS), no significant differences were observed in any measured parameters between the shaved group and the control group, indicating that shaving does not affect the reproductive performance of female rabbits under normal physiological conditions (Figures 1A–D). Under heat stress, the treatment group exhibited significantly higher numbers of offspring per litter and live births per litter compared to the control group, with statistically significance (^*^P < 0.05 for offspring, ^**^P < 0.01 for live births; Figures 1B, C). During the heat stress period, the body weight of newborn offspring in the treatment group was 404.159 ± 6.347 g, significantly higher than that in the control group (367.391 ± 7.317 g, ^**^P < 0.01). Similarly, during the recovery period, the body weight in the treatment group was 442.191 ± 6.237 g, significantly higher than that in the control group (406.242 ± 6.804 g, ^**^P < 0.01; Table 1). However, for individual birth weight, no significant differences were observed between the groups during either the heat stress or recovery periods (Figure 1D). These findings collectively demonstrate that shaving significantly enhances reproductive performance in pregnant female rabbits under heat stress, evidenced by improved offspring numbers, live births, and litter weight during and after heat stress exposure (Table 3, Figure 1).

**Table 3:** The influence of shaving on the reproductive performance of male and pregnant female rabbits at different periods of heat stress.

Period	Parameters	Control groups	Treatment groups
Pre-heat stress	Number of offspring per litter	8.406 ± 0.118 (*n* = 101)	8.262 ± 0.158 (*n* = 97)
	Number of live births per litter	8.109 ± 0.096 (*n* = 101)	8.062 ± 0.096 (*n* = 97)
	Newborn litter weight (g)	450.129 ± 6.347 (*n* = 101)	449.216 ± 6.233 (*n* = 97)
	Individual birth weight (g)	56.132 ± 0.650 (*n* = 101)	56.554 ± 0.687 (*n* = 97)
	Semen volume (ml)	1.23 ± 0.62 (*n* = 15)	1.18 ± 0.71 (*n* = 15)
	Sperm density (10^8^/ml)	5.29 ± 1.14 (*n* = 15)	5.43 ± 1.25 (*n* = 15)
	Sperm motility (%)	85.28 ± 4.52 (*n* = 15)	86.31 ± 4.48 (*n* = 15)
Heat stress	Number of offspring per litter	7.217 ± 0.151 (*n* = 46)	7.717 ± 0.163^*^ (*n* = 82)
	Number of live births per litter	6.957 ± 0.116 (*n* = 46)	7.512 ± 0.089^**^ (*n* = 82)
	Newborn litter weight (g)	367.391 ± 7.317 (*n* = 46)	404.159 ± 5.925^**^ (*n* = 82)
	Individual birth weight (g)	53.236 ± 1.134 (*n* = 46)	54.843 ± 0.307 (*n* = 82)
	Semen volume (ml)	0.82 ± 0.04 (*n* = 15)	0.99 ± 0.06 (*n* = 15)
	Sperm density (10^8^/ml)	2.57 ± 0.12 (*n* = 15)	3.14 ± 0.13^**^ (*n* = 15)
	Sperm motility (%)	65.53 ± 1.77 (*n* = 15)	73.23 ± 1.88^**^ (*n* = 15)
Recovery period	Number of offspring per litter	8.016 ± 0.131 (*n* = 62)	8.377 ± 0.169 (*n* = 89)
	Number of live births per litter	8.067 ± 0.105 (*n* = 62)	8.067 ± 0.105 (*n* = 89)
	Newborn litter weight (g)	406.242 ± 6.804 (*n* = 62)	442.191 ± 6.237^**^ (*n* = 89)
	Individual birth weight (g)	52.448 ± 0.650 (*n* = 62)	54.843 ± 0.307 (*n* = 89)
	Semen volume (ml)	0.77 ± 0.02 (*n* = 15)	0.93 ± 0.03^**^ (*n* = 15)
	Sperm density (10^8^/ml)	2.88 ± 0.12 (*n* = 15)	3.35 ± 0.15^*^ (*n* = 15)
	Sperm motility (%)	64.54 ± 1.10 (*n* = 15)	70.76 ± 1.32^**^ (*n* = 15)

Values are means ± SEMs.

^*^Values with different superscripts differ significantly (^*^p < 0.05; ^**^ *p* < 0.01).

### 3.3 The impact of shaving on metabolic activities in male bucks under normal condition

Since shaving itself constitutes a stressor for rabbits, its effects on metabolic activities under normal conditions need to be illustrated. To evaluate the impact of shaving on the reproductive performance of male rabbits under normal condition, metabolomic profiling and pathway enrichment analysis of seminal plasma from male rabbits were conducted.

**Figure 2:**
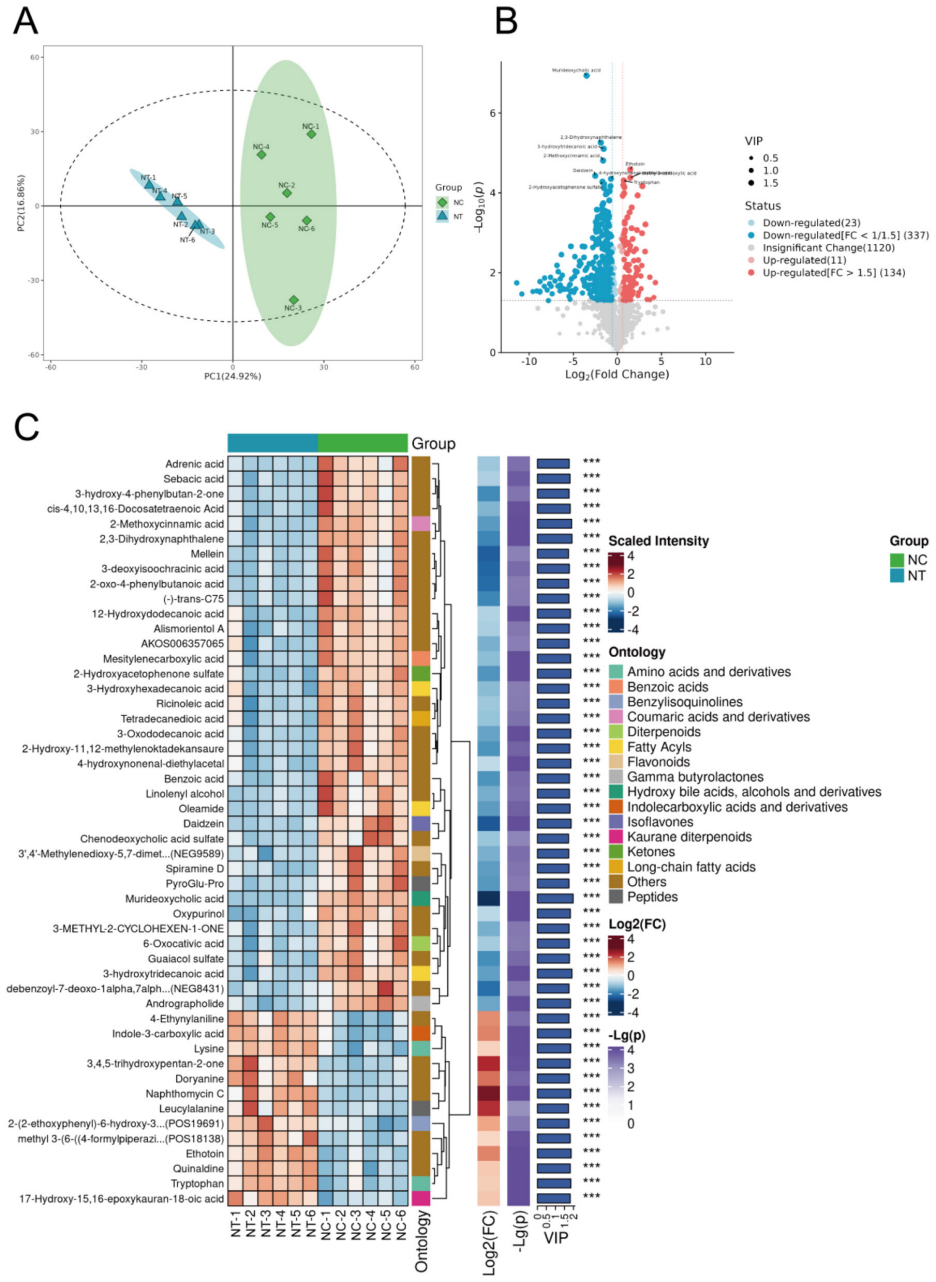
The effect of shaving on rabbit seminal plasma metabolites under normal conditions. **(A)** PCA (principal component analysis) plot shows the metabolic distribution differences between NC and NT groups, with green diamonds representing NC group samples (*n* = 6) and blue triangles representing NT group samples (*n* = 6). PC1: 24.92%, PC2: 16.86%. The confidence ellipses represent 95% confidence intervals for group clustering. **(B)** Volcano plot illustrates the expression changes of metabolites between NC and NT groups, with red dots indicating upregulated metabolites (FC > 1.5), blue dots indicating downregulated metabolites (FC < 1/1.5), and gray dots indicating no significant change, with some significant metabolite names and VIP values labeled. **(C)** Heatmap displays the abundance changes of different metabolite categories in NC and NT groups, with color gradients representing scaled intensity, and metabolite classifications and log_2_(FC) values listed on the right.

Principal component analysis (PCA) revealed distinct clustering between control groups under non-HS (NC) and treatment groups under non-HS (NT), indicating significant metabolic differences (Figure 2A). The volcano plot highlighted differentially expressed metabolites between the two groups, with variable importance in projection (VIP) scores highlighting their critical contributions to the observed differences (Figure 2B). Among the upregulated metabolites, ethotoin, indole-3-carboxylic acid, and tryptophan exhibited significant enrichment, whereas downregulated metabolites, including 2,3-dihydroxynaphthalene, 3-hydroxyhexadecanoic acid, and 2-methoxycinnamic acid, also demonstrated marked enrichment (Figure 2C).

**Figure 3:**
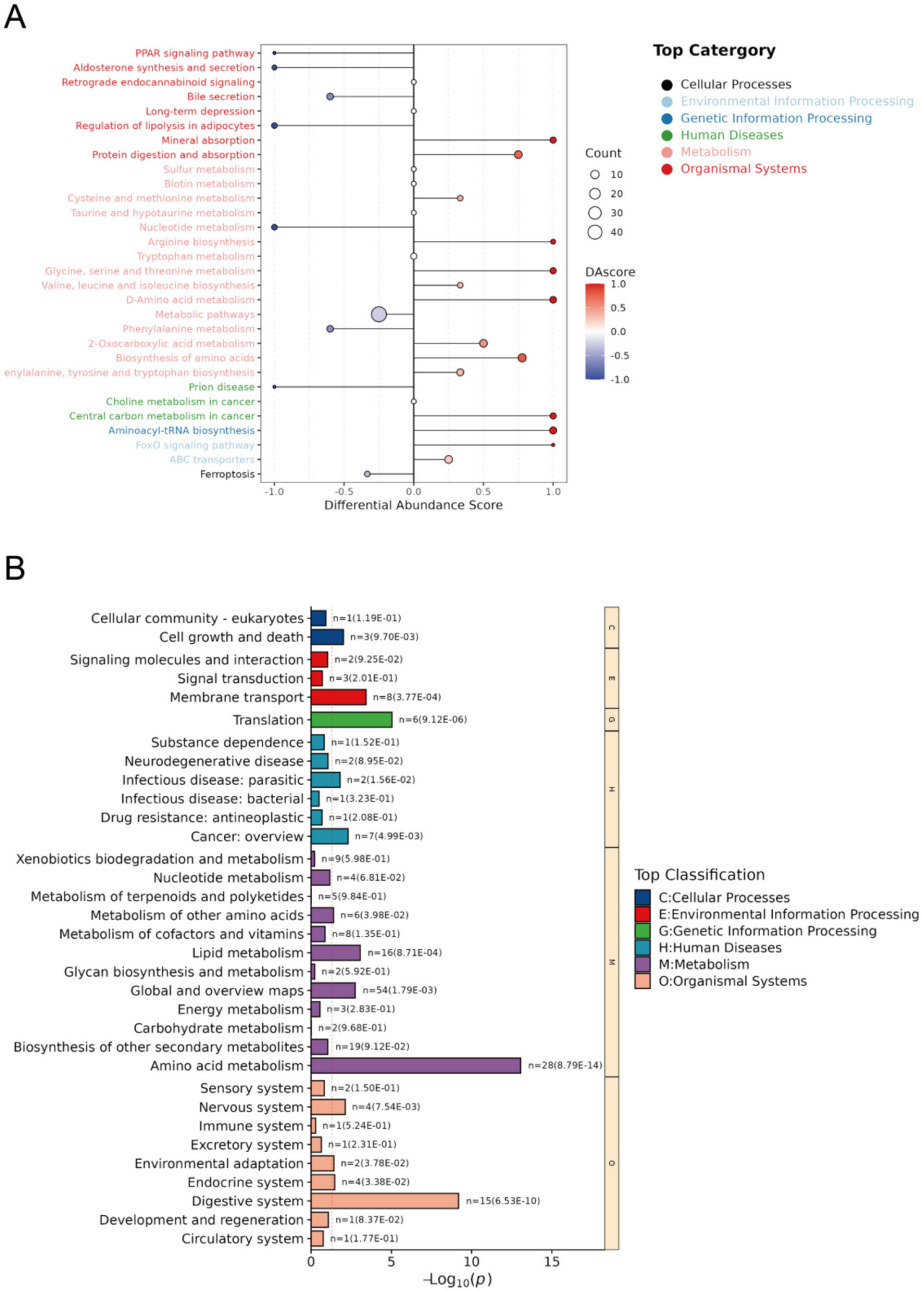
The effect of shaving on rabbit metabolic pathways under normal conditions. **(A)** Pathway enrichment analysis plot shows the differential abundance scores of various metabolic pathways, with point size indicating the number of metabolites in the pathway, color indicating the significance of enrichment, and the legend on the right displaying top categories. **(B)** Bar chart illustrates the enrichment significance (–log10(p) value) of each metabolic pathway, with colors distinguishing top categories, bar length indicating the number of metabolites, and sample counts and *p*-values for each category listed on the right.

KEGG analysis revealed that, differentially expressed metabolites were related to amino acid metabolism, digestive system, and translation (Figure 3A). Subsequent differential abundance (DA) score analysis revealed significant upregulation of pathways including aminoacyl-tRNA biosynthesis and D-amino acid metabolism (Figure 3B). Overall, multiple pathways within Amino acid metabolism were upregulated, consistent with prior findings, primarily glycine, serine and threonine metabolism and arginine biosynthesis, whereas phenylalanine metabolism was downregulated. Furthermore, regulation of lipolysis in adipocytes, aldosterone synthesis and secretion, and nucleotide metabolism exhibited significant downregulation.

### 3.4 The impact of shaving on metabolic activities in male rabbits under heat stress

**Figure 4:**
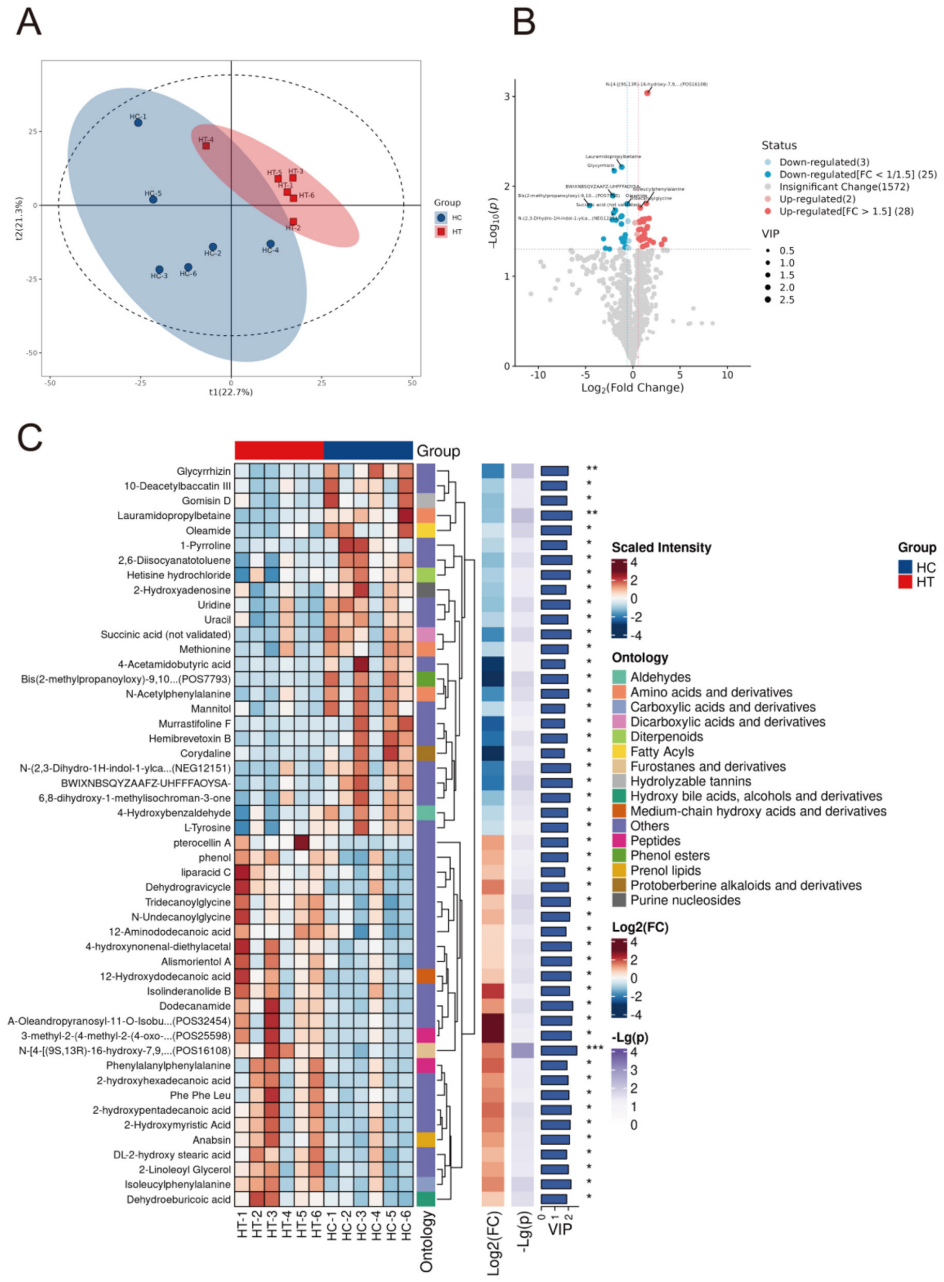
The effect of shaving on rabbit seminal plasma metabolites under heat stress conditions. **(A)** PCA plot shows the metabolic distribution differences between HC and HT groups, with blue circles representing HC group samples (*n* = 6) and red squares representing HT group samples (*n* = 6). T1: 22.7%, T2: 21.3%. The confidence ellipses represent 95% confidence intervals for group clustering. **(B)** Volcano plot illustrates the expression changes of metabolites between HC and HT groups, with red dots indicating upregulated metabolites (FC > 1.5), blue dots indicating downregulated metabolites (FC < 1/1.5), and gray dots indicating no significant change, with some significant metabolite names and VIP values labeled. **(C)** Heatmap displays the abundance changes of different metabolite categories in HC and HT groups, with color gradients representing scaled intensity, and metabolite classifications and log_2_(FC) values listed on the right.

To identify the key metabolites changed by shaving that affected the reproductive performance of male rabbits under HS, metabolomic profiling and pathway enrichment analysis of seminal plasma from male rabbits under HS were conducted. Principal component analysis (PCA) revealed distinct separation between control groups under HS (HC) and treatment groups under HS (HT) groups, with HC samples denoted by blue circles and HT samples by red squares (Figure 4A). This indicates significant metabolic differences induced by shaving under heat stress.

**Figure 5:**
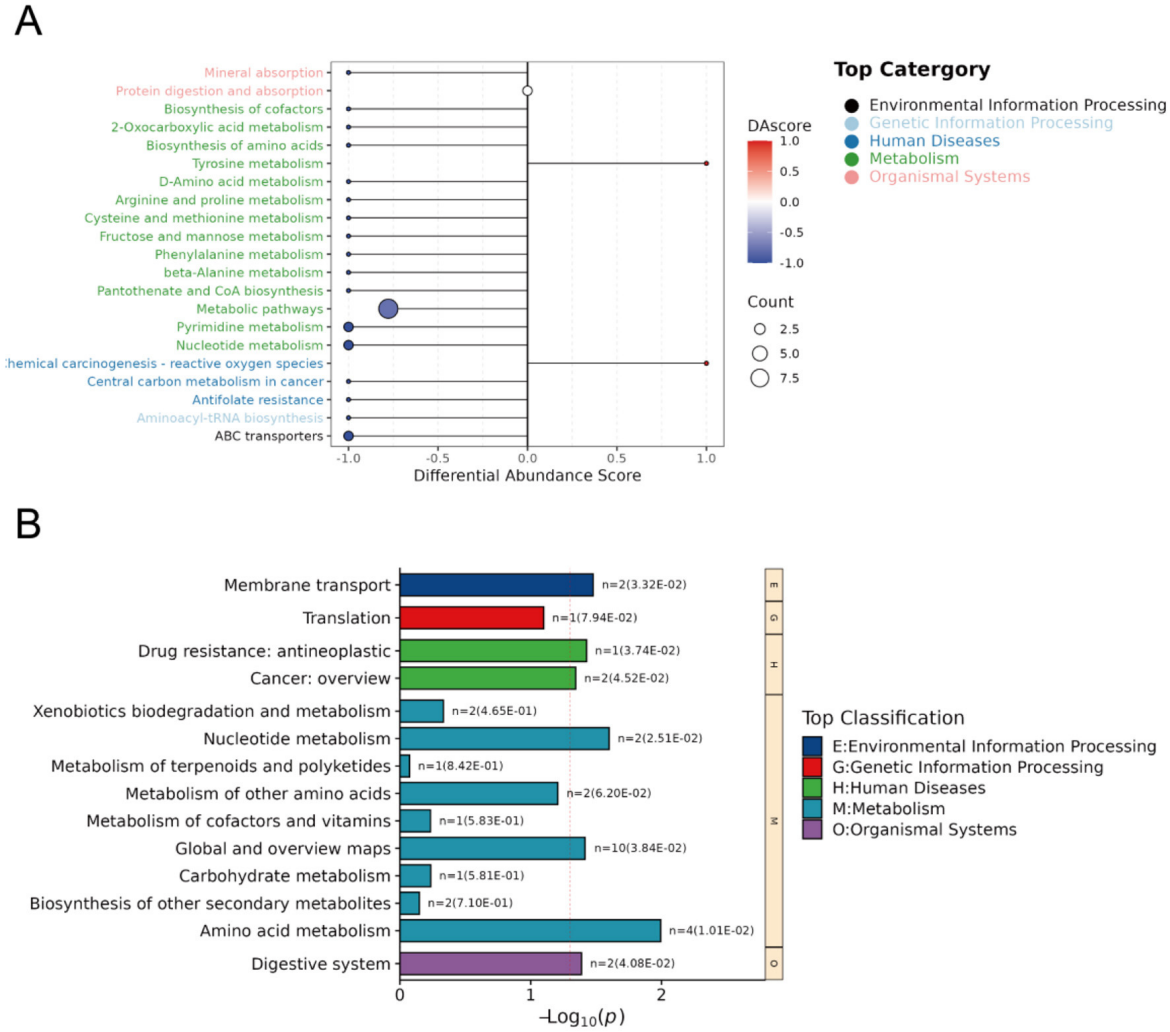
The effect of shaving on rabbit metabolic pathways under heat stress conditions. **(A)** Pathway enrichment analysis plot shows the differential abundance scores of various metabolic pathways, with point size indicating the number of metabolites in the pathway, color indicating the significance of enrichment, and the legend on the right displaying top categories. **(B)** Bar chart illustrates the enrichment significance (-log10(*p*) value) of each metabolic pathway, with colors distinguishing top categories, bar length indicating the number of metabolites, and sample counts and *p*-values for each category listed on the right.

The volcano plot (Figure 4B) revealed significant enrichment of isoleucylphenylalanine and tridecanoylglycine among the upregulated metabolites, whereas lauramidopropyl betaine, and glycyrrhizin showed marked enrichment among the downregulated metabolites. The heatmap illustrated abundance differences in metabolite categories between the HT and HC groups (Figure 4C). Overall, multiple pathways within amino acid metabolism were downregulated (Figure 5A)—a pattern opposite to that observed in the NT vs. NC comparison—predominantly D-amino acid metabolism, arginine and proline metabolism, cysteine and methionine metabolism, phenylalanine metabolism, and beta-alanine metabolism, whereas tyrosine metabolism was upregulated. Additionally, the chemical carcinogenesis–reactive oxygen species pathway exhibited significant upregulation (Figure 5B).

### 3.5 Identify the key metabolites participating in regulation of HS

**Figure 6:**
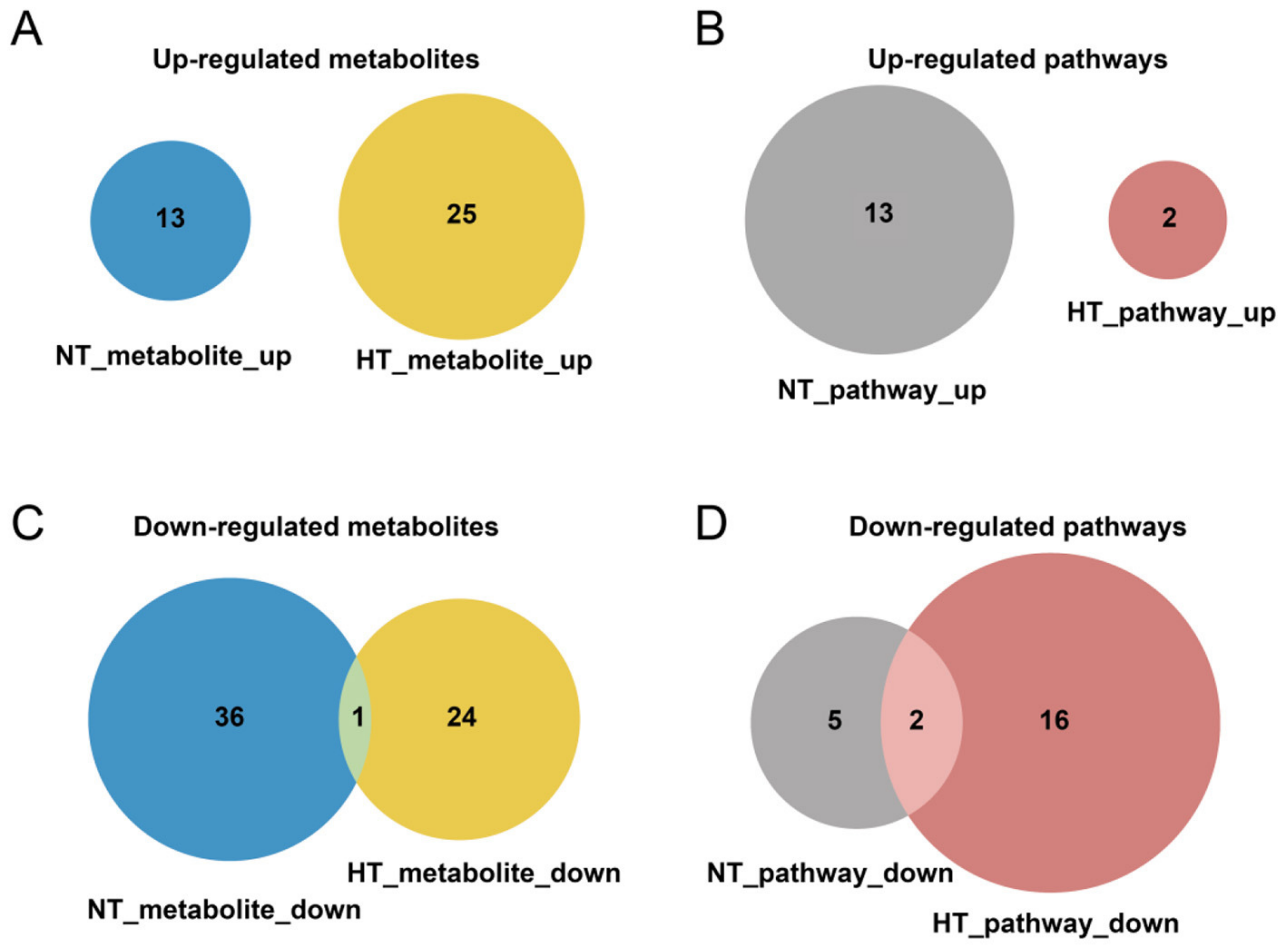
Venn diagram comparison of differential metabolites and pathways across different metabolic groups. **(A, B)** Up-regulated metabolites and pathways. **(C, D)** Down-regulated metabolites and pathways.

Among all upregulated differential metabolites and pathways induced by shaving, no shared metabolites or pathways were identified between the experimental group under normal physiological conditions and that under heat stress (Figures 6A, B). Among all downregulated differential metabolites induced by shaving, the NT and HT groups shared one common metabolite—oleamide, and two common pathways—nucleotide metabolism and phenylalanine metabolism (Figures 6C, D). This indicates that the downregulation of oleamide may contribute to adaptive responses to heat stress. In addition, nucleotide metabolism and phenylalanine metabolism might be the pathways that participated in regulating recovery of male reproduction after HS.

## 4 Discussion

Under HS conditions, semen quality parameters (semen volume, sperm density, and sperm motility) in the shaved group were significantly superior to those in the control group. Overall, during the 30-day recovery period, the shaved group exhibited accelerated semen quality recovery between RP-10 and RP-20, indicating that shaving not only alleviates the acute impacts of heat stress but also facilitates faster restoration of reproductive function. This may be attributable to reduced oxidative stress, since heat stress triggers excessive reactive oxygen species (ROS) production, impairing spermatogenesis via DNA damage and apoptosis (18, 19).

Under non-heat-stress conditions, seminal plasma metabolites from shaved and unshaved male rabbits exhibited significant separation (Figure 3A), indicating that shaving induced a systemic metabolic stress response. Differential metabolite analysis revealed upregulation of indole-3-carboxylic acid and tryptophan in the NT group, suggesting enhanced tryptophan metabolism. Indole-3-carboxylic acid, a metabolite derived from gut microbiota or hepatic tryptophan catabolism, may activate the aryl hydrocarbon receptor (AhR) pathway, potentially exerting antioxidant and anti-inflammatory effects, thereby mitigating the stress induced by shaving (20, 21). We observed downregulation of 2,3-dihydroxynaphthalene, 3-hydroxyhexadecanoic acid, and 2-methoxycinnamic acid. This may reflect a reduced reactive oxygen species (ROS) burden from polycyclic aromatic hydrocarbon metabolism (22) and suppression of the ω-oxidation pathway following lipid peroxidation (23), potentially indicating that the organism prioritizes energy homeostasis by minimizing oxidative stress sources and damage clearance burden. KEGG pathway enrichment analysis revealed overall upregulation of amino acid metabolism, with significant increases in the abundance of aminoacyl-tRNA biosynthesis, D-amino acid metabolism, glycine, serine and threonine metabolism, and arginine biosynthesis—pathways likely critical for protein synthesis during spermatogenesis. In contrast, downregulation of phenylalanine metabolism may reflect potential alterations in catecholamine-related pathways, thereby inhibiting regulation of lipolysis in adipocytes (24); suppression of nucleotide metabolism may represent an energy-conserving strategy to prioritize protein synthesis and germ cell proliferation for recovery from stress-induced impairments (25).

Under heat stress conditions, shaving treatment (HT vs. HC) similarly induced significant metabolic separation (Figure 4A), yet the metabolite profile differed markedly from that under normal conditions. We observed upregulation of isoleucylphenylalanine and tridecanoylglycine, which suggests accumulation of branched-chain amino acid-phenylalanine dipeptides and medium-chain acylglycines. This may potentially counteract elevated temperatures by enhancing antioxidant buffering or membrane stability (26). Conversely, downregulation of lauramidopropyl betaine and glycyrrhizin reflects depletion of surfactant-like metabolites and glycyrrhizic acid-class anti-inflammatory compounds, potentially indicating that shaving under heat stress failed to fully block the inflammatory cascade (27, 28). At the pathway level, amino acid metabolism was globally downregulated—opposite to the pattern observed under normal conditions—with particularly pronounced reductions in D-amino acid metabolism, arginine and proline metabolism, cysteine and methionine metabolism, phenylalanine metabolism, and beta-alanine metabolism. This may indicate depletion of the seminal plasma amino acid pool and limited energy availability for sperm. Conversely, tyrosine metabolism and the chemical carcinogenesis–reactive oxygen species pathway were upregulated, which may indicate that shaving under heat stress selectively activates the tyrosine–catecholamine axis and ROS scavenging systems to counter peak oxidative stress (29). We observed downregulation of nucleotide metabolism and ABC transporters, which may represent an adaptive response wherein, facilitated by shaving-induced cooling, the organism could suppress energy-intensive *de novo* nucleotide synthesis and non-essential transport processes. This potentially reallocates limited ATP to prioritize antioxidant defense and sperm motility maintenance, thereby promoting recovery of reproductive performance after heat stress.

Although the upregulated metabolites and pathways exhibited no overlap between the two conditions (Figure 6A), the downregulated profiles shared common elements, including oleamide, nucleotide metabolism, and phenylalanine metabolism (Figure 6B). Oleamide, an endogenous fatty acid amide with sleep-inducing and sedative properties, was concurrently downregulated in both the NT and HT groups. This may suggest that shaving could reduce overall stress burden by suppressing central–peripheral neurotransmitter-like signaling, thereby preserving reproductive resources in heat-stressed male rabbits (30). Downregulation of nucleotide metabolism was conserved across both conditions, which may indicate that shaving universally suppresses *de novo* nucleotide synthesis and purine/pyrimidine cycling, thereby minimizing energy expenditure and preventing oxidative stress-mediated DNA damage accumulation; this pathway's further suppression in heat stress, coupled with specific downregulation of pyrimidine metabolism, may amplify protective effects on sperm DNA integrity. We observed concurrent downregulation of phenylalanine metabolism, which may reveal that shaving limits the conversion of phenylalanine to tyrosine/catecholamines, preventing excessive sympathetic activation under heat stress that could lead to seminal vesicle vasoconstriction and reduced sperm motility; under normal conditions, this downregulation is consistent with phenylalanine homeostasis requirements during spermatogenesis.

The differentially expressed metabolites identified in this study were predominantly enriched in amino acid and nucleotide metabolism pathways. These pathways may play key roles in regulating sperm quality, and certain metabolites, such as oleamide and phenylalanine, may serve as potential biomarkers for the effectiveness of shaving in mitigating heat stress-induced declines in reproductive performance.

In female rabbits, shaving significantly improved reproductive performance under heat stress, with litter size, number of live-born pups, and neonatal litter weight being markedly higher than in the control group. During the recovery period, shaved females continued to exhibit significantly greater neonatal litter weight compared to controls. These findings align with prior studies indicating that heat stress disrupts oocyte maturation, embryonic development, and estrogen secretion, thereby reducing fertility. The absence of significant differences in individual birth weights suggests that shaving primarily enhances overall litter productivity by mitigating maternal heat stress, rather than directly influencing the growth potential of individual offspring. This may result from improved maternal thermoregulation via shaving, reduced energy expenditure on heat dissipation, and subsequent reallocation of resources to reproduction (31).

The findings of this study hold significant implications for rabbit husbandry, particularly during summer or in regions prone to high temperatures. Shaving, as a low-cost and non-invasive intervention, can be incorporated into rabbit management practices to improve reproductive performance and reduce economic losses caused by heat stress. The rapid recovery of semen quality in males and enhanced litter productivity in females demonstrate that shaving can increase overall population productivity.

Although this study provides compelling evidence for the benefits of shaving, several limitations persist. The experiments were conducted using bucks, thus, the generalizability of these findings to other breeds with different coat characteristics or thermoregulatory capacities remains unclear. Additionally, metabolomic analysis was confined to seminal plasma from male rabbits; extending such analyses to female reproductive tissues (e.g., ovary or uterus) could yield a more comprehensive understanding of shaving effects. The long-term effects of repeated shaving on rabbit health and hair regeneration require further investigation to ensure the sustainability of this practice in farming.

Future studies should investigate the molecular mechanisms underlying the observed metabolic alterations, particularly the roles of nucleotide metabolism, phenylalanine metabolism, and oleamide in stress modulation. Furthermore, integrating transcriptomic or proteomic analyses could clarify the interactions between metabolic pathways and gene expression under heat stress. Field trials in diverse climatic regions and across rabbit breeds are needed to further confirm the practical effectiveness of shaving as a heat stress mitigation strategy.

## 5 Conclusions

Summer shaving effectively alleviates heat stress in bucks, improving reproductive performance without compromising performance under thermoneutral conditions. In males, shaving increased semen volume, sperm density, and sperm motility. In females, it increased numbers of offspring per litter, live births per litter, and weight of newborn offspring under heat stress. Metabolomics revealed conserved downregulation of oleamide, nucleotide metabolism, and phenylalanine metabolism, thereby reducing oxidative stress and prioritizing reproductive resource allocation. Shaving provides a practical, low-cost strategy to improve rabbit productivity in hot climates.

## Data Availability

Metabolomics data generated in this study have been deposited in the MetaboLights database under accession number MTBLS15061 and are available at https://www.ebi.ac.uk/metabolights/MTBLS15061.
